# Achieving Equity in the Reach of Smoking Cessation Services Within the NCI Cancer Moonshot-Funded Cancer Center Cessation Initiative

**DOI:** 10.1089/heq.2020.0157

**Published:** 2021-06-16

**Authors:** Heather D'Angelo, Monica Webb Hooper, Jessica L. Burris, Betsy Rolland, Rob Adsit, Danielle Pauk, Marika Rosenblum, Michael C. Fiore, Timothy B. Baker

**Affiliations:** ^1^ Carbone Cancer Center, University of Wisconsin-Madison, Madison, Wisconsin, USA.; ^2^ Case Comprehensive Cancer Center, Case Western Reserve University, Cleveland, Ohio, USA.; ^3^ Markey Cancer Center, University of Kentucky, Lexington, Kentucky, USA.; ^4^ Department of Psychology, University of Kentucky, Lexington, Kentucky, USA.; ^5^ Institute for Clinical and Translational Research, University of Wisconsin-Madison, Madison, Wisconsin, USA.; ^6^ Center for Tobacco Research and Intervention, University of Wisconsin-Madison, Madison, Wisconsin, USA.

**Keywords:** cancer, smoking cessation, implementation science, tobacco-related health disparities

## Abstract

**Background:** Ensuring equitable access to smoking cessation services for cancer patients is necessary to avoid increasing disparities in tobacco use and cancer outcomes. In 2017, the Cancer Center Cessation Initiative (C3I) funded National Cancer Institute (NCI)-designated Cancer Centers to integrate evidence-based smoking cessation programs into cancer care. We describe the progress of C3I Cancer Centers in expanding the reach of cessation services across cancer populations.

**Methods:** Cancer centers (*n*=17) reported on program characteristics and reach (the proportion of smokers receiving evidence-based cessation treatment) for two 6-month periods. Reach was calculated overall and by patient gender, race, ethnicity, and age.

**Results:** Average reach increased from 18.5% to 25.6% over 1 year. Reach increased for all racial/ethnic groups, and in particular for American Indian/Alaska Native (6.6–24.7%), Asian/Native Hawaiian/Pacific Islander (7.3–19.4%), and black (18.8–25.9%) smokers. Smaller gains in reach were observed among Hispanic smokers (19.0–22.8%), but these were similar to gains among non-Hispanic smokers (18.9–23.9%). By age group, smokers aged 18–24 years (6.6–14.5%) and >65 years (16.1–24.5%) saw the greatest increases in reach.

**Conclusion:** C3I Cancer Centers achieved gains in providing smoking cessation services to cancer patients who smoke, thereby reducing disparities that had existed across important subgroups. Taking a population-based approach to integrating tobacco treatment into cancer care has potential to increase reach equity. Implementation strategies including targeted and proactive outreach to patients and interventions to increase providers' adoption of evidence-based smoking cessation treatment may advance reach even further.

## Introduction

Continuing to smoke after a cancer diagnosis results in poorer outcomes, including increased risk of a second primary cancer and decreased survival.^1–3^ Therefore, consistent tobacco use screening and timely referral to smoking cessation treatment is essential for improving patient outcomes.^4^ However, in practice, only about 60% of oncology clinicians report advising their patients to quit smoking and even fewer assist their patients in making quit attempts.^5^

To address this gap in quality cancer care, the National Cancer Institute (NCI) created the Cancer Center Cessation Initiative (C3I)^6^ and funded NCI-Designated Cancer Centers to create or expand evidence-based smoking cessation treatment programs.^7^ Cancer centers have increased the reach of smoking cessation treatment programs primarily by integrating treatment into clinical workflows through the electronic health record (EHR).^8–11^ However, given existing disparities in tobacco use, cessation treatment, and cancer treatment outcomes,^12,13^ it is important to ensure that cessation treatment programs are not only expanded, but equitable in their reach to all patients.

Tobacco-related disparities exist across demographic categories, such as race/ethnicity, age, and gender. Racial/ethnic minority populations experience a disproportionate burden of tobacco-related cancers,^13^ have lower smoking abstinence rates,^14^ and use cessation aids/pharmacotherapy at lower rates compared with white smokers.^15^ Multilevel factors, including discrimination, stress,^16^ exposure to environmental cues to smoke,^17^ and less access to smoking cessation treatment,^18^ play a role in these disparities. Disparities in access to cessation treatment may also exist as a function of age and gender. For example, females and adults age >65 years have been found to be prescribed cessation medications at lower rates,^19^ whereas males and younger adults are reached by quitline interventions at lower rates.^20^ However, the evidence is mixed with regard to gender in that some research suggests that women have equivalent or higher rates of treatment referral in primary care than do men^21,22^ and there is also evidence that women have equal or higher overall rates of smoking treatment engagement rates in population-based studies.^23,24^ Furthermore, younger adults are more likely to make unassisted versus assisted quit attempts,^15^ which are associated with lower abstinence rates.^25^

The C3I program encouraged health care system changes among participating cancer center programs in an effort to increase the reach of smoking treatment among all patients with cancer. This article examines how such changes were related to cessation program reach overall and to reach in different patient populations.

## Methods

The sample included 17 NCI-Designated Cancer Centers (“Centers”) that received C3I funding in October 2017; 5 Centers from the first cohort of C3I were excluded from this analysis because they did not provide the requisite data. Three of the 17 participating Centers implemented smoking cessation treatment programs in health care settings affiliated with the Cancer Center (e.g., community cancer hospital) for a total of 22 health care settings (2 Centers implemented in 3 settings; 1 Center implemented in 2 settings). Centers submitted completed questionnaires and data reports to the C3I Coordinating Center for two 6-month periods corresponding to July 1, 2018 to December 31, 2018 (“Time 1”) and January 1, 2019 to June 30, 2019 (“Time 2”). The study was deemed institutional review board (IRB) exempt and categorized as quality improvement and evaluation from the University of Wisconsin Madison.

For each 6-month period, Centers reported the cessation services offered (in-person counseling, telephone counseling, pharmacotherapy, referral to quitlines, referral to text/mobile or web-based programs, or interactive voice response systems), number of tobacco treatment specialists on staff, and whether they used their EHR to make referrals (“eReferral”). Centers submitted aggregate data extracted from the EHR for adults aged ≥18 years, including (1) total number of patients, (2) number of patients screened for smoking status, (3) number patients reporting current smoking (any past 30 days use of cigarettes), and (4) number of current smokers engaged in cessation treatment. Smoking status screening rates, current smoking prevalence, and cessation program reach were derived from the aggregate data. Reach was defined as the proportion of current smokers (denominator) that were engaged in at least one type of cessation treatment service (numerator). Engagement was broadly defined as attendance at any cessation counseling programs, including receiving brief advice to quit at the point-of-care, fax or EHR-referrals to quitlines, text/web programs, or receipt of pharmacotherapy. For each time period, reach was calculated for each setting overall and by demographics. Demographics included gender (male or female), race (American Indian or Alaska Native; Asian, Native Hawaiian or Pacific Islander; black or African American; white), ethnicity (Hispanic or non-Hispanic), and age group (18–24, 25–44, 45–64, and >65). Descriptive statistics were calculated across all settings using StataSE 16.0. Paired *t*-tests were used to compare differences in average reach at Time 1 and Time 2 (*α*=0.05) and results are presented as Supplementary Table S1.

## Results

### Changes in smoking cessation services

Table 1 shows the cessation services offered by time period. All 17 Centers offered in-person counseling by Time 2. Four Centers added telephone counseling, and two implemented an interactive voice response system to contact patients and engage them in cessation treatment. All Centers utilized eReferrals at Time 2, compared with 11 Centers that had this capability at Time 1. The number of Centers using automatic eReferrals to refer all identified smokers to cessation treatment doubled (from 4 to 8). The availability of both full- and part-time tobacco treatment specialists on staff also increased over time.

**Table 1.** Smoking Cessation Treatment Program Characteristics and Smoking Prevalence, Overall, and by Patient Demographics at National Cancer Institute-Designated Cancer Centers Within Cancer Center Cessation Initiative (*N*=17) Between 2018 and 2019

	Time 1	Time 2
**Cancer center level (*n*=17)**		
**Smoking cessation services**	***n* (%)**	***n* (%)**
In-person counseling	16 (94.1)	17 (100.0)
Telephone counseling	9 (52.9)	13 (76.5)
Pharmacotherapy	14 (82.4)	15 (88.2)
Quitline referrals	13 (76.4)	12 (70.6)
Text/web-based services referrals	7 (41.2)	9 (52.9)
Automated interactive voice response calls	0 (0.0)	2 (11.8)
EHR-based referrals to cessation	11(64.7)	17 (100.0)
Optional (provider/patient decides to use eReferral)	7 (41.2)	13 (76.4)
Automatic (all smokers referred)	4 (23.5)	8 (47.1)
Tobacco treatment specialists on staff		
1 or more part time	11 (64.7)	12 (70.6)
1 or more full time	7 (41.2)	10 (58.8)
**Health care setting level (*n*=22)**	**Mean (range)**	**Mean (range)**
Patients screened for smoking, (%)	86.2 (45.7–100.0)	89.1 (35.1–100.0)
Current smokers, (*n*)	1353 (71–3846)	1279 (41–3998)
Current smokers, (%)	10.9 (4.5–22.0)	11.4 (4.4–26.9)
Current smoker demographics (%)		
Gender		
Male	47.9 (32.4–58.6)	49.5 (36.1–62.4)
Female	51.9 (41.4–67.6)	50.5 (37.6–63.9)
Race		
American Indian or Alaska Native	0.7 (0.05–2.9)	0.6 (0.1–3.2)
Asian, Native Hawaiian or Pacific Islander	1.0 (0.2–5.2)	0.8 (0.1–3.1)
Black or African American	20.1 (1.7–63.5)	20.9 (2.2–58.7)
White	72.7 (34.6–95.8)	72.2 (35.0–95.1)
Ethnicity		
Hispanic	3.2 (0.6–9.7)	3.3 (0.3–9.2)
Non-Hispanic	92.6 (74.6–99.0)	92.6 (73.2–98.6)
Age		
18–24	1.5 (0.7–3.7)	1.2 (0.3–2.6)
25–44	15.9 (7.7–24.8)	15.4 (4.2–23.6)
45–64	54.0 (43.3–74.0)	52.8 (46.3–81.8)
65 and over	28.5 (15.5–38.9)	30.7 (14.0–40.3)

Time 1=July 1 to December 31, 2018; Time 2=January 1 to June 30, 2019.

EHR, electronic health record.

### Current smoking: screening and prevalence

Screening for smoking status changed from an average of 86.2% to 89.1% of patients screened (Table 1). Current smoking prevalence was stable across time and was, on average, about 11% across Centers. Demographic characteristics among current smokers were similar for both time periods. About half of patients reporting current smoking were female. An average of 72% of smokers were white, whereas about 20% were black or African American. American Indian or Alaska Native and Asian/Native Hawaiian/Pacific Islander smokers each represented 1% or less of the smokers at each Center, on average. An average of about 3% of smokers were identified as Hispanic. On average, a little over half of smokers were between the ages of 45 and 64 years, about 30% were ≥65 years, about 16% of smokers were between 25 and 44 years, whereas <2% were between 18 and 24 years.

### Changes in smoking treatment reach

**FIG. 1.**
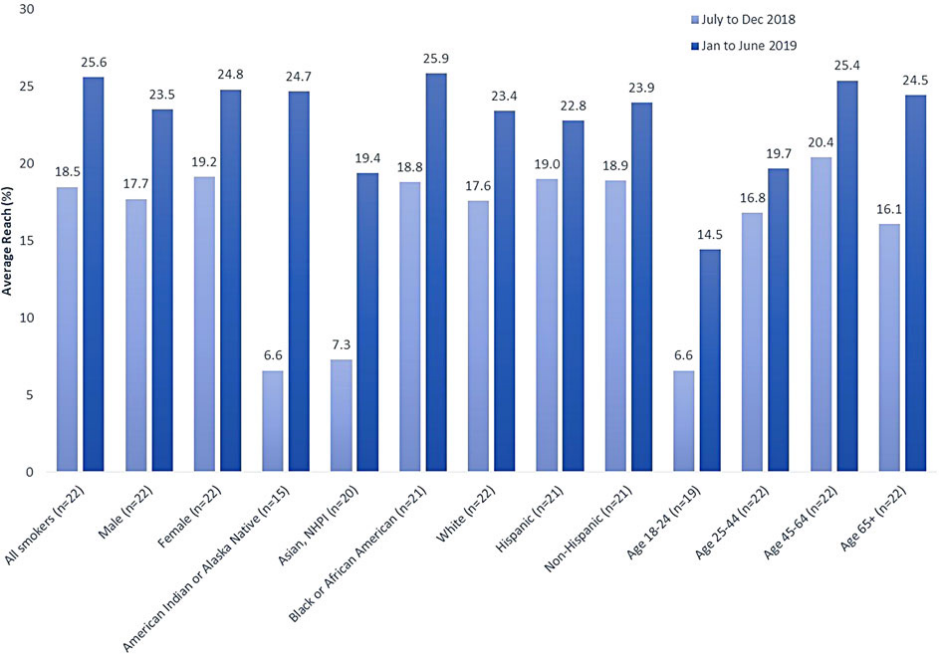
Change in average tobacco treatment program reach by patient demographics within NCI-designated Cancer Centers in the Cancer Center Cessation Initiative between July 1 to December 31, 2018 (Time 1) and January 1 to June 30, 2019 (Time 2). Reach is the percentage of smokers receiving tobacco treatment. Reach calculations required at least one smoker in the demographic group; therefore, the number of health care settings ranges from 15 to 22. NCI, National Cancer Institute; NHPI, Native Hawaiian or Pacific Islander.

Smoking cessation program reach increased from an average across settings of 18.5% of smokers receiving smoking cessation treatment at Time 1 to 25.6% of smokers at Time 2 (Fig. 1). There was variation in reach across settings, ranging from 2.4% to 80.6% of current smokers receiving at least one type of smoking cessation treatment (Supplementary Table S1). There was evidence of differential change in reach across time (Fig. 1). Although reach increased similarly for male and female patients, there was some evidence that reach increased differentially as a function of race. At Time 1, smoking treatment reach was lowest among those American Indian or Alaska Natives (6.6%) and Asian/Native Hawaiian/Pacific Islanders (7.3%) who smoked. At Time 2, average reach had increased to 24.7% of American Indian or Alaska Natives who smoked and 19.4% of Asian/Native Hawaiian/Pacific Islanders who smoked, increases on average of 18.1 and 12.1 percentage points, respectively (Supplementary Table S1). These increases decreased the gap in reach that had existed between these groups and the overall sample reach rate at Time 1. The difference observed for Asian/Native Hawaiian/Pacific Islanders reached statistical significance (*p*=0.04). Although the difference for American Indian/Alaska Natives did not reach significance (*p*=0.07), the increase in reach for American Indian/Alaska Natives essentially eliminated the disparity in reach for that group at Time 2. Reach at Time 1 was similar among black/African American (18.8%) and white (17.6%) individuals who smoked, and among Hispanic (19.0%) and non-Hispanic (18.1%) individuals who smoked. At Time 2, these groups had similar reach rates with the greatest gain in average reach occurring among black/African American smokers (mean difference=7.0, *p*=0.11). Reach differed meaningfully across age groups, with those aged 18–24 years having the lowest average reach at Time 1 (6.6%), and despite a 7.9-point increase, reach remained relatively low for this age group at Time 2 (14.5%). Reach increased among the 25–44 years age group, but only 19.7% of those who smoked in this age group were reached on average. Reach increased significantly for those age >65 years (mean difference 8.4, *p*=0.03). At Time 1, reach was only 16.1% among those age >65 years who smoked at Time 1, but this figure increased to 24.5% at Time 2, nearing the average reach among all individuals who smoked at that time.

## Discussion

The reach of smoking cessation treatment programs expanded among 17 NCI-funded C3I Cancer Centers over a 1-year period. This expansion was associated with the implementation of telephone-based counseling and text/web-based programs and the integration of EHR-based referral systems to connect patients directly with available cessation treatment programs. These structural changes may have contributed to increased reach by providing more flexible treatment options aligned with patient preferences, and by reducing barriers for oncology clinicians to connect patients with available cessation treatment or provide said treatment themselves.^26,27^

Some good news is that there was little evidence of reach inequities among several of the demographic groups assessed, with reach being largely equivalent at both Time 1 and 2 among black/African American, white, Hispanic, and non-Hispanic individuals. Although differences in reach among these groups did not achieve statistical significance, likely due to the low sample size of 22 settings, reach increased meaningfully. The gain in reach observed among black/African American individuals who smoked is notable and suggests that this group is interested and willing to engage in evidence-based care when it is offered and accessible. Furthermore, the preliminary reach inequities observed for some demographic groups and that were consistent with what is observed in the general population were reduced over time with the introduction of the enhanced smoking intervention systems changes. At Time 1, those individuals identifying as American Indian or Alaska Native were reached at far lower rates compared with all others who smoked at Time 1; that gap was not observed by Time 2. Significant gains in reach were also observed for Asian/Native Hawaiian/Pacific Islander individuals who smoked bringing them close to the average reach observed for the entire sample of those smoking at Time 2.

Among all groups of individuals who smoked at Time 1, reach was lowest among individuals aged 18–24 years. Reach more than doubled among this group at Time 2 but remained lower than the average reach rate for the entire sample. Substantial gains in reach were observed for those >65 years, indicating an opportunity to engage older smokers undergoing cancer treatment in smoking cessation treatment.

The current findings indicate that the implementation of a range of evidence-based smoking cessation treatments over a 1-year period, including brief cessation advice, pharmacotherapy, and referrals to quitlines, increased reach for many but not all demographic subgroups of individuals who smoked. It is important to bear in mind that reach reflects both patient willingness to engage in smoking treatment and clinician efforts to offer and deliver it. This could partly account for the differences in reach observed across some of the populations in this sample. Given the broad definition of reach used in this research, these findings indicate that more progress is needed to increase smoking treatment engagement among cancer patients overall, and especially among certain groups such as younger individuals and those >65 years.

Although expanding cessation services and streamlining referrals to treatment through the EHR is a promising strategy to increase reach generally, additional strategies such as population-wide proactive outreach to smokers across the continuum of readiness to quit^28^ may be needed to improve reach overall and to achieve equitable reach for certain subgroups of smokers in cancer care. Some subgroups of smokers might benefit from tailored culturally specific messaging about the benefits of smoking cessation and merits of treatment utilization.^29,30^ Internal quality improvement initiatives can identify multilevel barriers in the process of identifying and referring smokers to cessation treatment, and may help identify gaps in care.^31^ Provider-level barriers, including perceived time constraints to providing counseling, inadequate training or skills, or perceptions that cessation counseling is not efficacious may remain a barrier to reaching patients who smoke in some cancer care settings.^27,32^ Provider training and education, and audit and feedback measures have been shown to improve the reach of smoking cessation programs in cancer care, and implementation of these strategies could help achieve equity in reach.^9^

There are some limitations to this analysis. Results may not be representative of patients at all cancer centers, as this sample represents centers that received supplemental funding to support the enhancement or expansion of cessation services. However, smoking prevalence in this sample is similar to national estimates of current smoking among cancer survivors.^33,34^ Furthermore, <1% of the current smokers in this sample on average were American Indian or Alaska Natives and Asian/Native Hawaiian/Pacific Islanders, which may limit generalizability to these populations. Patient insurance status was inconsistently reported across Cancer Centers, and patient socioeconomic data (e.g., educational attainment, income level, and employment status) were not reported; therefore, reach across these sociodemographic domains could not be calculated. All data were collected in aggregate and, therefore, comparisons examining the intersection of groups were not possible (e.g., reach by gender and race). Staff workflow and education interventions for clinical staff are important for understanding program reach, but were not measured in this study. Despite these limitations, this is the first report to examine the reach across patient demographics among smoking cessation treatment programs implemented at NCI-Designated Cancer Centers.

In conclusion, the delivery of smoking cessation treatment to patients seen at NCI-Designated Cancer Centers as part of the C3I helped to expand cessation treatment services, increase overall reach and improve reach equity of those services. However, more work is needed to identify strategies to improve overall reach and equity in access to services among Asian/Native Hawaiian/Pacific Islander and younger smokers. Cancer Center leadership can play a key role in expanding the reach of smoking cessation treatment in cancer care by creating an organizational climate that is supportive of eliminating tobacco use among cancer patients, providing ongoing staff education to increase awareness of the importance of cessation and local cessation resources, and facilitating EHR and clinical workflow changes. Building connections between research, clinical, and administrative leadership is important for creating sustainable structural changes and attaining high levels of reach for all cancer patients who smoke.

## Supplementary Material

Supplemental data

## Abbreviations Used

C3I: Cancer Center Cessation Initiative
EHR: electronic health record
IRB: institutional review board
NCI: National Cancer Institute
NHPI: Native Hawaiian or Pacific Islander
