# Supplementary material for: Achieving Equity in the Reach of Smoking Cessation Services Within the NCI Cancer Moonshot-Funded Cancer Center Cessation Initiative
Source: Health Equity. 2021 Jun 16;5(1):424–30. doi: 10.1089/heq.2020.0157 (PMC8237098; doi:10.1089/heq.2020.0157)
Supplement: Supplemental data [file Supp_TableS1.docx]

|  | Supplemental Table 1. Smoking Cessation Program Reach at 17 C3I Cancer Centers by Patient Demographics | | | | | | | | | | |
| --- | --- | --- | --- | --- | --- | --- | --- | --- | --- | --- | --- |
|  | |  |  | | Smoking Cessation Program Reach (%) | | | | | | |
|  | |  | Time 1 (July to Dec 2018) | | | | Time 2 (Jan to June 2019) | | | Difference in mean (Time 2-Time 1) |  |
|  | | Number of settings | Mean | Min | | Max | Mean | Min | Max |  | p-value for paired t-test |
| All smokers | | 22 | 18.5 | 0.4 | | 87.3 | 25.6 | 2.4 | 80.6 | 7.1 | 0.09 |
| Sex | |  |  |  | |  |  |  |  |  |  |
| Male | | 22 | 17.7 | 0.3 | | 82.9 | 23.5 | 1.7 | 83.4 | 5.8 | 0.18 |
| Female | | 22 | 19.2 | 0.5 | | 91.7 | 24.8 | 2.2 | 76.7 | 5.7 | 0.17 |
| Race | |  |  |  | |  |  |  |  |  |  |
| American Indian or Alaska Native | | 15 | 6.6 | 0.0 | | 100 | 24.7 | 0.0 | 100 | 18.1 | 0.07 |
| Asian, Native Hawaiian or Pacific Islander | | 20 | 7.3 | 0.0 | | 33.3 | 19.4 | 0.0 | 100 | 12.1 | 0.04* |
| Black or African American | | 21 | 18.8 | 0.0 | | 83.1 | 25.9 | 1.4 | 88.5 | 7.0 | 0.11 |
| White | | 22 | 17.6 | 0.3 | | 86.8 | 23.4 | 2.07 | 80 | 5.8 | 0.16 |
| Ethnicity | |  |  |  | |  |  |  |  |  |  |
| Hispanic | | 21 | 19.0 | 0.0 | | 100.0 | 22.8 | 0.0 | 90 | 3.8 | 0.56 |
| Non-Hispanic | | 21 | 18.9 | 0.4 | | 86.8 | 23.9 | 1.99 | 80.1 | 5.0 | 0.26 |
| Age Group | |  |  |  | |  |  |  |  |  |  |
| 18-24 | | 19 | 6.6 | 0.0 | | 100 | 14.5 | 0.0 | 63.6 | 7.9 | 0.08 |
| 25-44 | | 22 | 16.8 | 0.0 | | 100 | 19.7 | 0.0 | 85.5 | 2.9 | 0.56 |
| 45-64 | | 22 | 20.4 | 0.3 | | 91.7 | 25.4 | 2.32 | 80.5 | 5.0 | 0.25 |
| 65+ | | 22 | 16.1 | 0.3 | | 72.7 | 24.5 | 1.82 | 78.2 | 8.4 | 0.03* |
| *P-value significance: *< 0.05 | | | | | | | | | | | |
